# Phylogenetic study of extirpated Korean leopard using mitochondrial DNA from an old skin specimen in South Korea

**DOI:** 10.7717/peerj.8900

**Published:** 2020-05-12

**Authors:** Jee Yun Hyun, Jang Hyuk Cho, Puneet Pandey, Mi-Sook Min, Kyung Seok Kim, Hang Lee

**Affiliations:** 1 Conservation Genome Resource Bank for Korean Wildlife (CGRB), Research Institute for Veterinary Science and College of Veterinary Medicine, Seoul National University, Seoul, Republic of Korea; 2 Tiger and Leopard Conservation Fund in Korea, Seoul, Republic of Korea; 3 Amity Institute of Forestry and Wildlife, Amity University, Uttar Pradesh, India; 4 Department of Natural Resource Ecology and Management, Iowa State University, Ames, IA, USA

**Keywords:** Korean leopard, South Korea, Old skin, Mitochondrial DNA, *Panthera pardus* orientalis

## Abstract

The leopard, *Panthera pardus*, is a threatened species in its range throughout the world. Although, historically, the Korean Peninsula had a high population density of leopards, they were extirpated from South Korea by 1970, leaving almost no genetic specimens. Traditionally, Korean leopards are classified as *Panthera pardus orientalis*; however, their classification is based only on locality and morphology. Therefore, there is a need for genetic studies to identify the phylogenetic status of Korean leopards at the subspecies level. Presently, no extant wild specimen is available from South Korea; therefore, we extracted genetic material from the old skin of a leopard captured in Jirisan, South Korea in the 1930s and conducted the first phylogenetic study of the South Korean leopard. A total of 726 bp of mitochondrial DNA, including segments of the NADH5 and control region, were amplified by PCR. A phylogenetic analysis of the fragment, along with sequences of nine leopard subspecies from GenBank revealed that the extinct South Korean leopard belonged to the Asian leopard group and in the same clade as the Amur leopard (*Panthera pardus orientalis*). Thus, the leopard that inhabited South Korea in the past was of the same subspecies as the Amur leopard population currently inhabiting the transboundary region of Russia, China, and North Korea. These results emphasize the importance of conserving the endangered wild Amur leopard population (estimated to be about 60–80 individuals) in Russia and China, for future restoration of leopards in the Korean Peninsula.

## Introduction

The leopard, *Panthera pardus*, is a generalist carnivore among the large cat species and was historically distributed throughout a large part of the world (Nowell & Jackson, 1996; Jacobson et al., 2016). The leopard population has undergone a rampant decrease over the years and is on the brink of extinction in many regions due to human threats such as overhunting, poaching and habitat loss and fragmentation (Jacobson et al., 2016). Leopards were once found throughout the Korean peninsula in the past and were sometimes referred to as the Korean leopard (Ognev, 1962; World Wildlife Fund (WWF), 2019).

Throughout Asia, leopards were considered harmful animals that threatened public safety and their skins were mainly collected as tributes and trophies in the past (Jacobson et al., 2016; Jo & Baccus, 2016). These animals were systematically captured over a long period of time, from the Joseon dynasty (1392–1910) through the Japanese colonial period (1910–1945). The annual number captured decreased rapidly in the early 20th century and eventually, they were difficult to find in the Korean peninsula by the end of the Japanese colonial period (Jo & Baccus, 2016). Historical records indicate that at least 624 leopards were killed under the policy of eliminating harmful animals during the Japanese occupation (Governor-General of Korea, 1917, 1926, 1942; Endo, 2009, 2014). There have been informal eyewitness accounts of leopards until the 2000s (Jo, 2008; Nam, 2012), but formally the last wild leopard in South Korea was captured and photographed in Yeohangsan in 1970 (Chosun Ilbo, 1970; Kyunghyang Shinmun, 1970). The International Union for Conservation of Nature (IUCN) considers that the leopard has been extinct in South Korea and estimates the extinction occurred after 1969 but the exact time is not know because tracks have been seen after that (Nowell & Jackson, 1996; Stein et al., 2016).

The tiger is another large feline species that was also extirpated from South Korea. A phylogenetic study of Korean tigers was conducted using skull and bone specimens from Japan’s National Science Museum and the Smithsonian Museum in the USA (Lee et al., 2012). Hides are a common form of preservation of mammalian specimens, both in museum collections and as trophies (Hedmark & Ellegren, 2005). Thus, specimens from museums and private collections might be available for DNA analysis of extinct carnivores. Unfortunately, when the leopard became extinct in South Korea, there were almost no remains left of the leopard from which genetic specimens could be extracted. However, we discovered an old leopard skin in South Korea and herein present a phylogenetic analysis using DNA samples extracted from this specimen.

There are nine subspecies of leopards in the world (Miththapala, Seidensticker & O’Brien, 1996; Uphyrkina et al., 2001). Recently, phylogenetic relationships of the leopards have been refined by analyzing additional samples from previously uncovered areas (Rozhnov, Lukarevskiy & Sorokin, 2011; Farhadinia et al., 2015; Wilting et al., 2016). However, genetic studies on the subspecific classification of the historical leopard population in South Korea have not yet been performed. Such information is vital for formulating restoration and management strategies aimed at restoring or reviving extirpated species in a region. A previous study on the leopard subspecific phylogeny used only North Korean samples (Uphyrkina et al., 2001) and genome research conducted in South Korea used an Amur leopard sample from a zoo (Kim et al., 2016). Although leopards in South Korea are classified as the Amur leopard subspecies *P. pardus orientalis* (Jacobson et al., 2016), their classification is based only on sample locality and morphology information. Therefore, identification of the phylogenetic status of Korean leopards based on genetic studies is important to document the subspecific status of leopards in South Korea.

In this study, we obtained genetic material from the old skin of a leopard captured in Jirisan, South Korea in the 1930s and conducted a phylogenetic study to identify the subspecific status of leopards in South Korea, an important historical habitat of this large carnivore.

## Materials and Methods

### Sample collection

**Figure 1:**
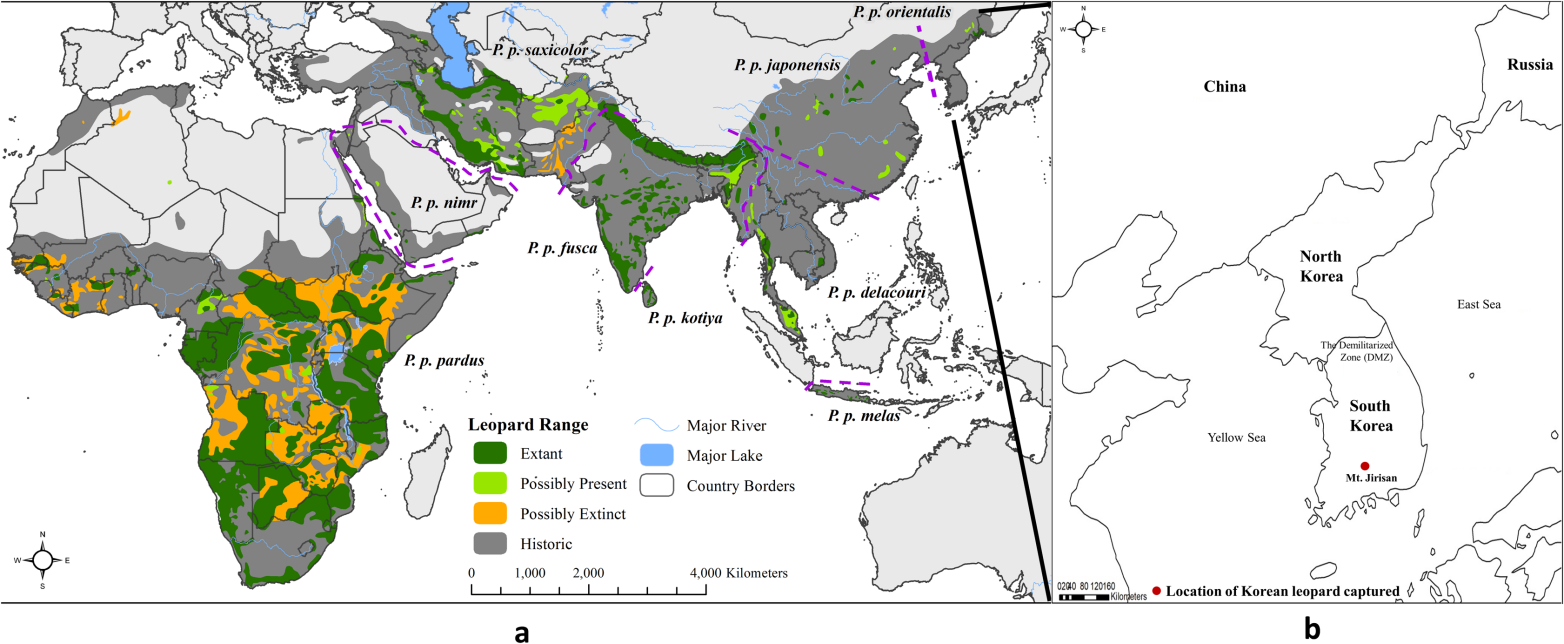
Distribution map of leopard subspecies and sample information. (A) Distribution of leopard subspecies (source: Jacobson et al., 2016). (B) Capture location of leopard specimen used in this study.

Due to the limited South Korean leopard samples left, we could collect only one specimen. Although limited in quantity, it might be a suitable specimen to determine the phylogenetic position of Korean leopards since the specimen was originated from the southern tip of the Korean Peninsula (Fig. 1). The sample was donated to and stored in Conservation Genome Resource Bank for Korean Wildlife (CGRB) at Seoul National University, Republic of Korea (http://www.cgrb.org). The registration number was CGRB15834.

The specimen was a skin rug of a Korean leopard caught by a Korean hunter in Jirisan, South Korea, in 1935 during the period of Japanese occupation. Genetic samples were collected from this skin rug. Considering the difficulty of extracting DNA from old skins (Moraes-Barros & Morgante, 2007), we sampled various sites of the leopard specimen, including the foot pad, claw, ear and nose. Only the internal portions of each part were collected to avoid any contamination due to external exposure during long-term storage.

### DNA extraction, polymerase chain reaction and sequencing

DNA was extracted using the QIAGEN QIAamp DNeasy Blood & Tissue Kit (Qiagen, Hilden, Germany). Initially, we performed PCR amplification using the leopard species-specific primer set Ppo_CbF/Ppo_CbR (Ppo_CbF: 5′-GTAAATTATGGCTGAATTATCCGG-3′, Ppo_CbR: 5′-CATAACCGTGAACAATAATACGAC-3′) (Sugimoto et al., 2006) to amplify a 156 bp DNA fragment of cytochrome *b*, to check the success of DNA extraction from the old skin.

Three primer sets were used for PCR amplification of two segments of mitochondrial DNA (total of 726 bp), including the NADH dehydrogenase subunit 5 (NADH5) gene (611 bp) and control region (CR) gene (115 bp). We used two primer sets, F/RL2 and F2/RL4 (F: 5′-GTGCAACTCCAAATAAAAG-3′, RL2: 5′-TAAACAGTTGGAACAGGTT-3′, FL2: 5′-CGTTACATGATCGATCATAG-3′, RL4: 5′-TTAGGTTTTCGTGTTGGGT-3′) (Uphyrkina et al., 2001), each of which amplified approximately 400 bp of the NADH5 mitochondrial gene. In the Uphyrkina et al. (2001) study, there was not enough information on the primers for the control region, so we designed a primer set CR_PP_F/CR_PP_R (CR_PP_F: 5′-CCTCAACTGCCCGAAA-3′, CR_PP_R: 5′-CATGGCCCTGAAGTAAGAA-3′) to amplify the targeted 115 bp segment of the CR gene.

The PCR included a final reaction volume of 15 μL containing 10× PCR buffer (iNtRON Biotechnology, Inc., Gyeonggi-do, South Korea), 0.2 mM dNTP, 0.75 μM of each primer, 9 μg of bovine serum albumin (BSA), 0.375 units of Taq DNA polymerase (iNtRON Biotechnology, Inc., Gyeonggi-do, South Korea), and three μL of template DNA. PCR cycling conditions were as follows: 5 min initial denaturation at 94 °C; 45 cycles of 0.5 min denaturation at 94 °C, 0.5 min annealing at 56 °C for cyt *b* or 53 °C for CR and 45 s extension at 72 °C; and a final extension for 10 min at 72 °C. Reaction conditions for the NADH5 gene were as follows: 5 min initial denaturation at 94 °C; 45 cycles of 0.5 min denaturation at 94 °C, 1.5 min annealing at 50 °C, 1 min extension at 72 °C; and a final extension for 10 min at 72 °C. PCR was conducted on a TaKaRa PCR thermal cycler dice (TaKaRa, Tokyo, Japan). A total of three μL of the PCR products were electrophoresed on 1% agarose gel in 0.5 X Tris–borate–EDTA (TBE) buffer. Purification was performed using a Zymoclean Gel DNA Recovery kit (Zymo Research, Irvine, CA, USA). Each step of experimentation included a negative control to prevent contamination and a positive control of a tissue sample from a naturally dead leopard in a zoo to ensure adequate conditions for successful experiments. The NADH5 and CR segments were sequenced in both forward and reverse directions using the BigDye Cycle Sequencing Kit version 3.1 on an ABI 3700xl sequencer analyzer (Applied Biosystems^™^, Foster City, CA, USA). DNA sequence data of the Korean leopard were deposited into GenBank (accession number: MK114159–MK114160).

### Data analysis

The final nucleotide sequence of each NADH5 and CR segment was determined by the assembly of both direction sequences using Geneious Pro 5.3 software (Kearse et al., 2012). For the phylogenetic analysis, representative haplotype sequences of all leopard subspecies (Uphyrkina et al., 2001; Wilting et al., 2016) were downloaded from NCBI (accession numbers AY03522–AY035292 and JN811043–JN811046). For an outgroup, sequences from *Panthera tigris* (KJ508412–KJ508413) were used. The nucleotide sequences of the Korean leopard, nine subspecies of leopard, and the tigers were aligned together using the Geneious alignment option implemented in Geneious Pro v 5.3 (Kearse et al., 2012) (http://www.geneious.com). Concatenated sequences (726 bp) of NADH5 and CR for all samples were used for phylogenetic tree construction.

Phylogenetic trees were constructed by maximum likelihood (ML) analysis in MEGA 6.0 (Tamura et al., 2013) with 10,000 bootstrap replicates and the HKY + G + I model of sequence evolution following a Bayesian Information Criterion (BIC) model of selection (File S1). MrBayes v3.2 (Ronquist & Huelsenbeck, 2003) and BEAST v1.7.4 (Drummond et al., 2012) were also used to construct phylogenetic trees using Bayesian inference (BI) with the HKY + G + I model, which was found to be the most suitable using the jModeltest 2.1.10 software. In BEAST, Bayesian analysis was conducted using an uncorrelated lognormal relaxed clock model and Markov Chain Monte Carlo (MCMC) chain lengths of 10,000,000 iterations and every 1,000th tree was logged. The Figtree v1.3.1 software was used to visualize the trees (http://tree.bio.ed.ac.uk/software/figtree). Network analysis for the mtDNA sequence data was conducted using the NETWORK v5.0.0.3 software package (Bandelt, Forster & Rohl, 1999).

## Results

In this study, DNA from an old skin of the Korean leopard was successfully extracted and targeted fragments of NADH5, CR and cytochrome *b* were amplified from mitochondrial DNA. The primer Ppo_Cb successfully amplified the cytochrome *b* fragment from DNA extracted from the inside of the claw, ear and foot pad. However, the relatively long fragment (~400 bp) of the NADH5 region was amplified only from the DNA extracted from the claw. A comparison of the concatenated nucleotide sequences of the Korean leopard and nine leopard subspecies revealed a new haplotype (KOR1) in the Korean leopard. Among the nine leopard subspecies, the South Korean leopard showed the highest genetic similarity with two haplotypes of Amur leopard (ORI1, ORI2) from North Korea and Russia. KOR1 differed from ORI2 in only two nucleotide sites—one in the NADH5 segment (position 189 of the concatenated sequences) and the other in the CR segment (position 725 of the concatenated sequences) (Table 1).

**Table 1:** Polymorphic sites of mitochondrial NADH5 and control region (CR) partial genes of leopards (*Panthera pardus*) from South Korea and position number is from the beginning of concatenated sequences of NADH5 and CR.

Nucleotide	NADH5 (611 bp)																																
Position	9	10	12	18	23	25	33	39	44	59	64	71	77	89	125	139	149	152	156	164	167	170	183	189	209	212	215	221	242	251	254	262	266
KOR1	C	C	T	T	T	T	G	T	T	C	T	C	A	T	T	T	C	G	A	C	T	C	A	G	A	A	T	C	T	T	C-	T	C
ORI1	–	–	–	–	–	–	–	–	–	–	–	–	–	–	–	–	–	–	–	–	–	–	–	A	–	–	–	–	–	–	–	–	–
ORI2	–	–	–	–	–	–	–	–	–	–	–	–	–	–	–	–	–	–	–	–	–	–	–	A	–	–	–	–	–	–	–	–	–
JAP1	–	T	–	–	–	–	–	–	–	–	–	–	–	–	–	–	–	–	–	–	–	–	–	–	–	–	–	–	–	–	–	C	–
JAP2	–	T	–	–	–	–	–	–	–	–	–	–	–	–	–	–	–	–	–	–	–	–	–	–	–	–	–	–	–	–	–	–	–
DEL1	–	T	–	C	–	C	–	–	–	–	–	–	–	–	–	–	–	–	–	–	–	–	–	–	–	–	–	–	–	–	–	–	–
DEL2	–	T	–	–	–	C	–	–	–	–	–	–	–	–	–	–	–	–	–	–	–	–	–	–	–	–	–	–	–	–	–	–	–
DEL3	–	T	–	C	–	C	–	–	–	–	–	–	–	–	–	–	–	–	–	–	–	–	–	–	–	–	–	–	–	–	–	–	–
KOT1	–	T	–	–	C	–	–	–	–	–	–	–	–	–	–	–	–	–	–	–	–	–	–	T	–	–	–	–	–	–	–	C	–
KOT2	–	T	–	–	C	–	–	–	–	–	–	–	–	–	–	–	–	–	–	–	–	–	–	T	–	–	–	–	–	–	–	C	–
KOT3	–	T	–	–	C	–	–	–	–	–	–	–	–	–	–	–	–	–	–	–	–	–	–	T	–	–	–	–	–	–	–	C	–
FUS1	–	T	–	–	–	–	–	–	–	–	–	T	–	C	–	–	–	–	–	–	–	–	–	T	–	–	–	–	–	–	–	–	–
FUS2	–	T	–	–	–	–	–	–	–	–	–	–	–	C	–	–	–	–	–	–	–	–	–	T	–	–	–	–	–	–	–	–	–
FUS3	–	T	–	–	–	–	–	–	–	–	–	–	–	–	–	–	–	–	–	–	–	–	–	T	–	–	–	–	–	–	–	–	–
FUS4	–	T	–	–	–	–	–	–	–	–	–	–	–	–	–	–	–	–	–	–	–	–	–	T	–	–	–	–	–	–	–	–	–
FUS5	–	T	–	–	–	–	–	–	–	–	–	–	–	–	–	–	–	–	–	–	–	–	–	T	–	–	–	–	–	–	–	–	–
FUS6	–	T	–	–	–	–	–	–	–	–	–	–	–	–	–	–	–	–	–	–	–	–	–	T	–	–	–	–	–	–	–	–	–
SIN1	–	T	C	–	–	–	–	–	–	–	–	–	–	–	–	–	–	–	–	–	–	–	–	T	–	–	–	–	–	–	–	–	–
SAX1	–	T	C	–	–	–	–	–	–	–	–	–	–	–	–	–	–	–	–	–	–	–	–	T	–	–	–	–	–	–	–	–	–
SAX2	–	T	–	–	–	–	–	–	–	–	–	–	–	–	–	–	–	–	–	–	–	–	–	T	–	–	–	–	–	–	–	–	–
NIM1	–	T	–	–	–	–	–	–	–	–	–	–	G	C	–	–	T	–	–	–	–	–	–	T	–	–	–	–	C	–	T	–	–
SHO1	–	T	–	–	–	–	–	–	C	–	–	–	–	C	–	–	T	A	–	–	M	–	G	T	–	T	C	–	–	–	T	–	–
SHO2	–	T	–	–	–	–	–	–	Y	–	–	–	–	C	–	–	T	R	–	–	M	–	G	T	–	T	C	–	–	–	T	–	–
SHO3	–	T	–	–	–	–	–	–	–	–	–	–	–	C	–	–	T	–	–	–	C	–	G	T	–	T	C	–	–	–	T	–	–
SHO4	–	T	–	–	–	–	–	–	–	–	–	–	–	C	–	–	T	–	–	–	C	–	G	T	–	T	C	–	–	–	T	–	–
SHO5	–	T	–	–	–	–	–	–	–	–	–	–	–	C	C	–	T	–	–	–	C	–	G	T	–	T	C	–	–	–	T	–	–
SHO6	–	T	–	–	–	–	–	–	–	T	–	–	–	C	–	C	T	–	–	T	C	–	G	T	–	T	C	–	–	–	T	–	–
SHO7	–	T	–	–	–	–	–	–	–	–	–	–	–	C	–	–	T	–	–	–	C	–	G	T	–	T	C	–	–	–	T	–	–
SHO8	–	T	–	–	–	–	–	–	–	–	–	–	–	C	–	–	T	–	G	–	C	–	G	T	–	T	C	–	–	–	T	–	T
SHO9	–	T	–	–	–	–	A	–	–	–	C	–	–	C	–	–	T	–	–	–	A	–	–	–	–	–	–	–	–	–	T	–	–
SHO10	–	T	–	–	–	–	A	–	–	–	C	–	–	C	–	–	T	–	–	–	A	–	–	–	–	–	–	–	–	–	T	–	–
SHO11	–	T	–	–	–	–	A	–	–	–	C	–	–	C	–	–	T	–	–	–	A	–	–	–	–	–	–	–	–	–	T	–	–
SHO12	–	T	–	–	–	–	A	–	–	–	C	–	–	C	–	–	T	–	–	–	A	–	–	–	–	–	–	–	–	–	T	–	–
MEL1	–	T	–	–	–	–	–	C	–	–	–	–	–	C	–	–	T	–	–	–	T	T	–	–	–	–	–	T	–	C	–	–	–
MEL2	–	T	–	–	C	–	–	C	–	–	–	–	–	C	–	–	T	–	–	–	T	T	–	–	–	–	–	T	–	C	–	–	–
MEL3	–	T	–	C	–	–	–	C	–	–	–	–	–	C	–	–	T	–	–	–	T	T	–	–	G	–	–	T	–	C	–	–	–
MEL4	T	T	–	–	–	–	–	C	–	–	–	–	–	C	–	–	T	–	–	–	T	T	–	–	–	–	–	T	–	C	–	–	–
MEL5	–	T	–	–	–	–	–	C	–	–	–	–	–	C	–	–	T	–	–	–	T	T	–	–	–	–	–	–	–	C	–	–	–

Nucleotide	NADH5 (611 bp)																											Control region (115 bp)						
Position	272	278	281	296	305	320	323	329	353	383	407	437	440	449	452	461	473	479	500	533	539	545	548	553	573	575	611*	621	629	633	634	651	693	725
KOR1	C	A	G	A	A	A	C	C	T	T	C	A	A	T	A	A	A	T	C	C	T	A	T	C	G	T	T	A	C	T	A	G	A	T
ORI1	–	–	–	–	–	–	–	–	–	–	–	–	–	C	–	–	–	–	–	–	–	–	–	–	A	C	–	–	–	–	–	–	–	C
ORI2	–	–	–	–	–	–	–	–	–	–	–	–	–	–	–	–	–	–	–	–	–	–	–	–	–	–	–	–	–	–	–	–	–	C
JAP1	–	–	–	–	–	–	–	–	–	–	–	–	–	C	–	–	–	–	–	–	–	–	–	–	–	C	–	–	–	–	–	–	–	C
JAP2	–	–	–	–	–	–	–	–	–	–	Y	–	–	C	–	R	–	–	–	–	–	–	C	–	–	–	–	–	–	–	–	–	–	C
DEL1	–	–	–	–	–	–	–	–	–	–	–	–	–	C	G	–	–	–	–	–	–	–	–	–	–	–	C	–	–	–	–	–	G	C
DEL2	–	–	–	–	–	–	T	–	–	–	–	–	–	C	G	–	–	–	–	–	–	–	–	–	–	–	C	–	–	–	–	–	–	C
DEL3	–	–	–	–	–	–	–	–	–	–	–	–	–	C	–	–	–	–	–	–	–	–	–	–	A	C	–	–	–	–	–	–	G	C
KOT1	–	–	–	–	–	–	–	–	–	–	–	–	–	C	–	–	–	–	–	–	–	–	–	–	–	–	–	–	–	C	–	A	–	C
KOT2	–	–	–	–	–	–	–	–	–	–	–	–	–	–	–	–	–	–	–	–	–	–	–	–	–	–	–	–	–	C	–	A	–	C
KOT3	–	–	–	–	–	–	–	–	–	–	–	–	–	–	–	–	–	–	–	–	–	–	–	–	–	–	–	–	–	–	–	A	–	C
FUS1	–	G	–	–	–	–	–	–	C	C	Y	–	–	C	–	–	R	–	–	Y	–	–	–	–	–	–	–	–	–	–	–	A	–	C
FUS2	–	G	–	–	–	–	–	–	C	C	T	–	–	C	–	–	–	–	–	–	–	–	C	–	–	–	–	–	–	–	–	A	–	C
FUS3	–	G	–	–	–	R	–	–	C	C	T	–	–	C	–	–	G	–	T	T	C	–	–	–	–	–	–	–	–	–	–	A	–	C
FUS4	–	G	–	–	–	–	–	–	Y	–	Y	–	–	C	–	–	G	C	T	T	C	–	–	–	–	–	–	–	–	–	–	A	–	C
FUS5	–	G	–	–	–	–	–	–	T	–	–	–	–	C	–	–	–	–	–	–	–	–	–	T	–	–	–	–	–	–	–	A	–	C
FUS6	–	G	–	–	–	–	–	–	K	–	–	–	–	–	–	–	–	–	–	–	–	–	–	–	–	–	–	–	–	–	–	A	–	C
SIN1	–	–	–	–	–	–	–	–	–	–	–	–	–	C	–	–	–	–	–	–	–	–	–	–	A	–	–	–	–	–	C	A	–	C
SAX1	–	–	–	–	–	–	–	–	–	–	–	–	–	C	–	–	–	–	–	–	–	–	–	–	A	–	–	–	–	–	–	A	–	C
SAX2	–	–	–	–	–	–	–	–	–	–	–	–	–	C	–	–	–	–	–	–	–	–	–	–	–	–	–	–	–	–	–	A	–	C
NIM1	–	–	A	G	G	G	–	T	C	–	T	G	–	C	–	–	–	–	–	–	–	–	–	–	–	–	–	–	–	–	–	A	–	C
SHO1	–	–	A	G	–	G	–	–	C	C	T	–	–	C	–	–	G	–	T	T	–	–	–	–	–	–	–	–	T	C	–	A	–	C
SHO2	–	–	A	G	–	G	–	–	G	C	T	–	–	C	–	–	G	–	T	T	–	–	–	–	–	–	–	–	–	C	–	A	–	C
SHO3	–	–	A	G	–	G	–	–	S	C	T	–	–	C	–	–	G	C	T	T	C	–	–	–	–	–	–	–	T	C	–	A	–	C
SHO4	–	–	A	G	–	G	–	–	C	C	T	–	–	C	–	–	G	C	T	T	C	–	–	–	–	–	–	–	–	G	–	A	G	C
SHO5	–	–	A	G		R	–	–	C	C	T	–	–	C	–	–	G	–	T	T	–	–	–	–	–	–	–	G	T	C	–	A	–	C
SHO6	–	–	A	G	–	G	–	–	C	C	–	–	–	C	–	–	–	–	–	–	–	T	–	–	–	–	–	G	T	C	–	A	–	C
SHO7	–	–	A	G	–	G	–	–	G	–	–	–	–	C	–	–	–	–	–	–	–	–	–	T	–	–	–	–	T	C	–	A	–	C
SHO8	–	–	A	G	–	R	–	–	K	–	–	–	–	C	–	–	–	–	–	–	–	–	–	–	A	–	–	–	–	C	–	A	–	C
SHO9	T	–	A	G	–	G	–	–	C	–	–	–	–	C	–	–	–	–	–	–	–	–	–	–	A	C	–	–	–	C	–	A	–	C
SHO10	T	–	A	G	–	G	–	–	C	–	–	–	–	C	–	–	–	–	–	–	–	–	–	–	A	C	–	–	–	–	–	A	G	C
SHO11	T	–	A	G	–	G	–	–	C	C	T	–	–	C	–	G	–	–	–	–	–	–	C	–	–	–	–	–	–	–	–	A	G	C
SHO12	T	–	A	G	–	G	–	–	C	–	–	–	G	C	–	G	–	–	–	–	–	T	–	–	–	–	–	–	–	–	–	A	G	C
MEL1	–	–	A	–	–	–	–	–	–	C	–	–	–	C	–	–	–	–	–	–	–	–	–	–	–	–	–	–	–	–	–	A	G	C
MEL2	–	–	A	–	–	–	–	–	–	C	–	–	–	C	–	–	–	–	–	–	–	–	–	–	–	–	–	–	–	–	–	A	G	C
MEL3	–	–	A	–	–	–	–	–	–	C	–	–	–	C	–	–	–	–	–	–	–	–	–	–	–	–	–	–	–	–	–	A	G	C
MEL4	–	–	A	–	–	–	–	–	–	C	–	–	–	C	–	–	–	–	–	–	–	–	–	–	–	–	–	–	–	–	–	A	G	C
MEL5	–	–	A	–	–	–	–	–	–	C	–	–	–	C	–	–	–	–	–	–	–	–	–	–	–	–	–	–	–	–	–	A	G	C

**Notes:**

KOR, Korean leopard; ORI, *P. p. orientalis;* JAP, *P. p. japonensis;* DEL, *P. p. delacouri;* KOT, *P. p. kotiya;* FUS, *P. p. fusca;* SAX, *P. p. saxicolor;* NIM, *P. p. nimr;* SHO, *P. p. pardus;* MEL, *P. p. melas;* A, adenine; C, cytosine; G, guanine; T, thymine; R, G or A; Y, T or C; K, G or T; M, A or C; S, G or C.

* The last number of NADH5 region.

**Figure 2:**
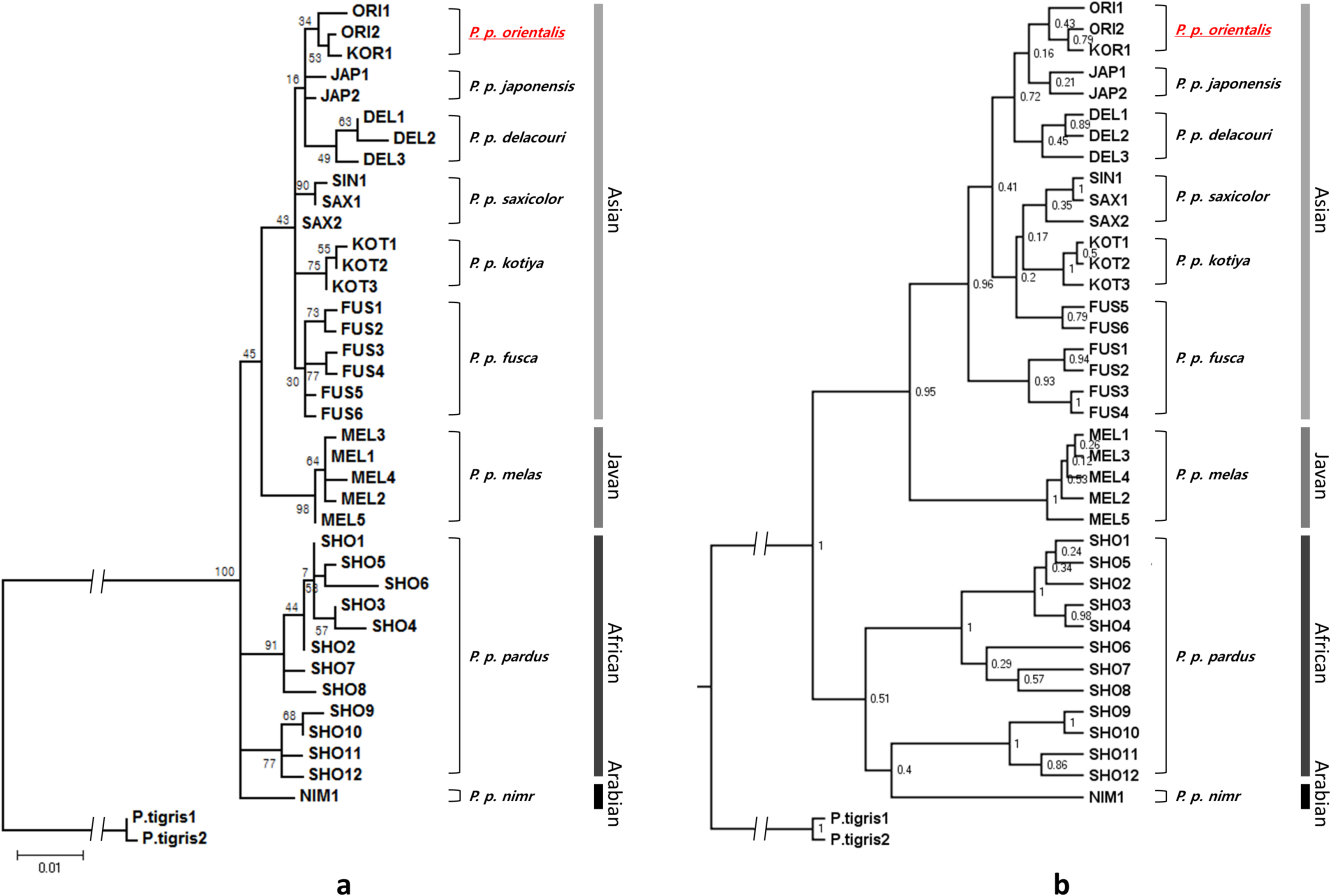
Phylogenetic relationships among the leopard mtDNA haplotypes from combined NADH5 and CR mitochondrial regions. Individual samples of *Panthera tigris* are taken as outgroup species. (A) Maximum likelihood (ML) tree. ML tree approach performed using HKY + G + I model. (B) Bayesian tree constructed using by Beast with HKY + G + I model. KOR, Korean leopard; ORI, *P. p. orientalis;* JAP, *P. p. japonensis;* DEL, *P. p. delacouri;* KOT, *P. p. kotiya;* FUS, *P. p. fusca;* SAX, *P. p. saxicolor;* NIM, *P. p. nimr;* SHO, *P. p. pardus;* MEL, *P. p. melas*.

The phylogenetic relationships among the leopard subspecies were similar among ML trees. The ML tree supported the division of leopards into nine subspecies, except that the North Chinese leopards (JAP) and Persian leopards (SAX) were not grouped into a single clade. The African leopard (SHO) was divided into two sub-groups. The Korean leopard was grouped into the Amur leopard (ORI) lineage (Fig. 2A). Three subspecies (ORI, JAP and DEL) with ranges in the eastern and southeastern parts of continental Asia were grouped together, including the Korean and Amur leopards. This clade was included in the larger Asian leopard group. MEL from Java was more closely related to the Asian group than the African.

We used two independent software packages to construct the Bayesian tree using the same nucleotide substitution. The tree topology obtained using MrBayes was not consistent with the geographical distribution (File S2) of defined leopard subspecies. The results of the Bayesian phylogenic analysis using BEAST clustered different subspecies into its own subclade and are shown in Fig. 2B. The leopard subspecies formed three distinct clusters corresponding to Asian, Javan and African + Arabian distributions. Among the Asian leopards, the Korean leopards (KOR1) and Amur leopards (ORI1 and ORI2) were grouped into a single clade. In general, the Korean leopard belonged to the Asian group and formed the same lineage as the Amur leopard in both ML and Bayesian trees with support values of 53% (bootstrap) and 0.79 (posterior probability), respectively (Fig. 2).

**Figure 3:**
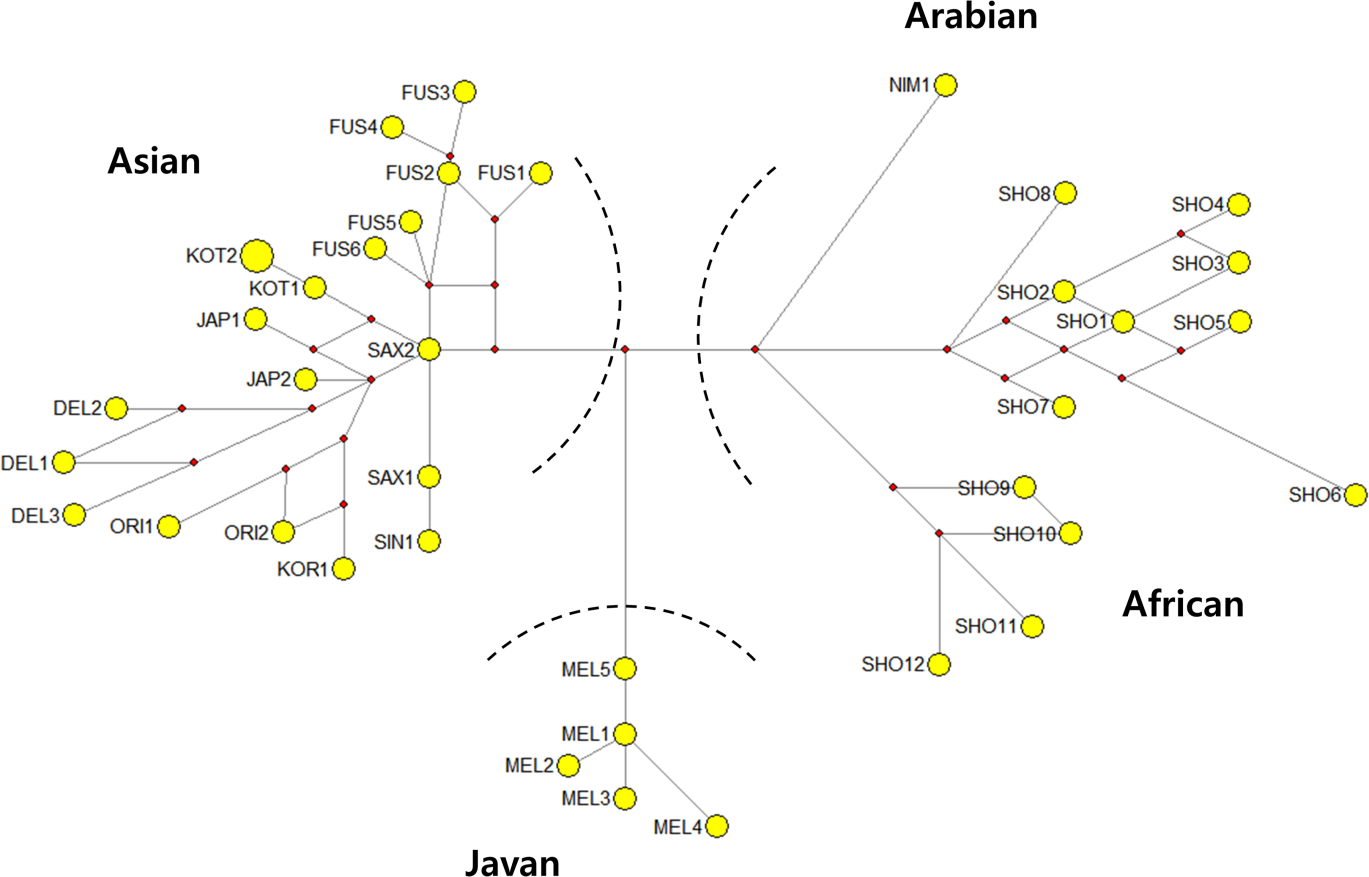
Median-joining network showing the relationships among leopard mtDNA haplotypes, based on 726 bp sequence of the mtDNA NADH5 and CR gene. KOR, Korean leopard; ORI, *P. p. orientalis;* JAP, *P. p. japonensis;* DEL, *P. p. delacouri;* KOT, *P. p. kotiya*, FUS, *P. p. fusca;* SAX, *P. p. saxicolor;* NIM, *P. p. nimr;* SHO, *P. p. pardus;* MEL, *P. p. melas*.

Network analysis revealed that Asian, Javan, and African + Arabian leopard groups constituted three major clusters (Fig. 3). Samples from each subspecies within the Asian group were grouped together according to geographic proximity (ORI, DEL, FUS, JAP, KOT and SAX). The network analysis was in agreement with the results from the phylogenetic tree reconstructions.

## Discussion

It is difficult to extract DNA from ancient samples due to risk of loss from degradation and breakage (Hall, Willcox & Jones, 1997; Pääbo et al., 2004; Moraes-Barros & Morgante, 2007). DNA extraction from the claws of carnivorous trophy skins is most efficient for genetic studies (Hedmark & Ellegren, 2005). In this study, DNA was also extracted successfully from the claws of the South Korean leopard hide and more than 600 base pairs of the mitochondrial DNA were amplified.

### Phylogenetic status of Korean leopard

Phylogenetic reconstructions in this study demonstrated that leopards are divided into three major groups, corresponding to Asian, Javan, and African + Arabian distributions. This result is similar to recent studies on leopard phylogeny (Wilting et al., 2016), where the authors suggested classifying leopards into three groups. More specifically, looking into the BI, the leopard subspecies formed two major clusters: (1) larger Asian group and Javan and (2) African and Arabian. ML and BI topology are similar in this study and they both supported well the nine subspecies divided in the previous study (Uphyrkina et al., 2001). The position of the Arabian leopard (NIM) is slightly different between ML and BI, which was also reported in previous studies (Farhadinia et al., 2015; Uphyrkina et al., 2001; Wilting et al., 2016). Sometimes it was included within the African clade and at other times, it was independent of Africa. These conflicting results might be due to a low resolution caused by short length of sequences and small sample size and further study is therefore required to resolve this issue.

All phylogenetic trees showed that the South Korean leopard was tied to the same lineage as the Amur leopard in the Asian group. In the Asian group, clades reflected subspecific classifications, except for JAP. The clades of three subspecies (ORI, DEL, JAP), which are distributed geographically from eastern to southeastern Asia, were observed as one cluster in the ML and BI phylogeny. The Amur leopard to which the South Korean leopard belongs, was clustered with the Asian group and the East Southern Asian group. Leopard subspecies that are distributed geographically more closely in East and Southeast Asia are genetically closer (Fig. 1). Although the clustering pattern of some Asian subspecies is not clear in the ML and BI tree to distinguish the subspecies (JAP, FUS and SAX), the tree topology clearly assigned the South Korean leopard (KOR) and Amur leopard (ORI) to the same clade.

In our study, it was difficult to clearly distinguish subspecies of Asian leopards because branch lengths were short. A possible reason for this may be that the information contained in the target sequence used in the analysis was insufficient to classify the subspecies. Recently, the Cat Specialist Group of IUCN/SSC suggested that there are eight subspecies of leopard, excluding *P. p. japonensis* which was merged with *P. p. orientalis* (Kitchener et al., 2017). Regardless, in the phylogenic trees from both the ML and Bayesian analyses of this study, the Korean leopard was placed in the same lineage as the Amur leopard. The results of present genetic study is in agreement with the general expectation that there is no biogeographic barrier between the Northeast Asian Continent and Korean Peninsula that may inhibit dispersal of leopards and gene flow between the populations in these regions. Therefore, we propose that the Korean leopard and the Amur leopard belong to the same subspecies and is part of the Asian group. However, such interpretation needs to be taken with a caution since the phylogenetic relationship was explored based on a single specimen as a potential Korean leopard and larger sample sizes would reveal a level of variation among sequences from different individuals. Given the limited sample size in this study, a comprehensive study using more samples would be useful in clarifying subspecific status of Korean leopard within the Asian group.

### Conservation and restoration of the Korean leopard

The present results suggest possible directions for the future restoration of leopards in the Korean Peninsula. There seem to be two ways to restore the leopard population in the Korean Peninsula. One possible way is the reintroduction of leopards into the South Korean region using captive-bred Amur leopard individuals. A similar proposal has been made to reintroduce leopards into the southern Sikhote-Alin region (Kelly, Stack & Harley, 2013; Hebblewhite et al., 2011). Previous studies suggest that the reintroduction of leopards, which are relatively safer than tigers, might be possible and the best area for this would be Kangwon province, which includes the Korea demilitarized zone (DMZ) (Jeon, Lee & Kang, 2009; Lim, 2017). Amur leopards use smaller home ranges (33–139 km^2^ for a female leopard) than Amur tigers (384 km^2^ for tigress) and are nonexclusive (Salmanova, 2008; Goodrich et al., 2010; Lee et al., 2013; Spitzen et al., 2013). In addition, due to their smaller body size and lower energy requirements, Amur leopards are expected to be in less conflict with humans than Amur tigers (Goodrich et al., 2011; Spitzen et al., 2013). For the initial reintroduction of leopards, previous studies proposed the area near the eastern part of the DMZ because of its low human population density and high density of prey animals such as ungulates (Choi, 2005; Jeon et al., 2008; Jeon, Lee & Kang, 2009; National Institute of Environmental Research, 2013; Choi, 2015). The DMZ region is a part of the major ecological network of the Korean Peninsula. This area consists of high mountains and a dense temperate forest, which are important habitats for wildlife (Lim, 2017).

IUCN guidelines for reintroduction highlight the importance of sourcing founders from genetically close populations (IUCN, 1998). Based on our findings that the extinct Korean leopard and the Amur leopard belong to the same subspecies, the Amur leopard is an ideal candidate for a restoration project. We propose to consider carefully planning a reintroduction program for leopards in South Korea using individuals from a captive population of Amur leopard currently managed globally within the Amur Leopard Global Species Management Plan (GSMP), coordinated by the World Association of Zoos and Aquariums (World Association of Zoos and Aquariums (WAZA), 2019). The Amur leopard GSMP manages four regional captive populations (EAZA, EARAZA, AZA and JAZA) globally and 209 individual leopards descended from 13 founders distributed in 88 institutions in the world as of April 2013. The current size of the global wild population of Amur leopards is estimated to be less than 100 individuals (Vitkalova et al., 2018), and this number is too small to be utilized for translocation for a reintroduction program. Therefore, the translocation of wild individuals is not currently an option for a potential reintroduction program. However, since the global captive populations of Amur leopard GSMP are scientifically managed based on conservation genetics principles and metapopulation theory (Leus, Traylor-Holzer & Lacy, 2011), and the captive Amur leopard population belongs to the same genetic lineage as the historical leopard population that inhabited the Korean Peninsula, the GSMP population is well suited to a future reintroduction program in South Korea. The potential reintroduction program could be designed following the reintroduction strategy developed to increase wild Amur leopard populations in the Russian Far East as a model (Kelly, Stack & Harley, 2013). Rigorous assessments of potential habitat, human-leopard conflict issues and disease risks, in addition to the social and financial support needed for success, will be essential components of developing a viable reintroduction program (Hebblewhite et al., 2011; Kelly, Stack & Harley, 2013).

Another way of leopard restoration in the Korean Peninsula is through the natural dispersal of leopard individuals into North Korean territory. The size of the wild Amur leopard population in the Russian-Chinese-North Korean transboundary region has increased rapidly in recent years from approximately 30 to 80, thanks to the intensive cooperative conservation efforts between China and Russia. The Land of the Leopard National Park in the very southern part of Primorsky Krai, Russia serves as the main stable habitat supporting the global increase in leopard population (Jackson & Nowell, 2008; Sugimoto et al., 2014; Xiao et al., 2014; Qi et al., 2015; Vitkalova et al., 2018). Natural dispersal of individuals from this growing wild leopard population may result in range expansion of leopards into North Korean territory in the northern part of the Korean peninsula. Intimate cooperation among North Korea, China and Russia to implement habitat and population conservation measures will be essential for the natural restoration and maintenance of a stable leopard population in North Korea.

## Conclusions

South Korea is in the process of restoring endangered wildlife such as Asiatic black bears, gorals and foxes (Ministry of Environment in Korea, 2010), and conducted a basic study on the possibility of the restoration of tigers in Korea (Lee et al., 2013). Efforts to restore endangered species in many parts of the world are underway, including the Amoy tiger in China, the cheetah and lion in Africa, the brown bear and lynx in Europe, the wolf in Canada, the cougar and black bear in the USA, and so on (Wotschikowsky & Kerger, 1990; Breitenmoser et al., 1999, 2014; William & Robert, 2004; Ueda, 2008). In Russia, besides the Land of the Leopard National Park, a restoration project is underway to reintroduce the Amur leopard into the Lazo Nature Reserve, a former habitat of leopards (Christie, Cook & Arzhanova, 2012; Spitzen et al., 2012; Kelly, Stack & Harley, 2013). Like these, in situations where humans are endeavoring to restore endangered species, phylogenetic studies are the fundamental step and important. For successful restoration of endangered species, the first task should be to clarify the taxonomic status of that species. For example, genetic studies were carried out to confirm the phylogenetic position and genetic diversity of reintroduced individuals in the case of the Asiatic black bear, which has been successfully restored in Korea (Kim et al., 2011). Our phylogenetic study suggested that the extinct leopards formerly occupying the Korean peninsula belong to the Amur leopard subspecies (*P. pardus orientalis*) currently inhabiting Far East Russia.

Based on the results of this study, we suggest introducing Amur leopard pedigree individuals when attempting to reintroduce leopards into South Korea in the future. The restoration of the leopard in North Korea can be expected through the natural influx of wild Amur leopard populations across the Far East Russia-China-North Korea border. However, an eco-corridor may be needed to facilitate this natural range expansion. Above all, the Amur leopard in the wild is still a critically endangered subspecies, with only 60–80 individuals worldwide (Jackson & Nowell, 2008; Sugimoto et al., 2014; Xiao et al., 2014; Qi et al., 2015; Vitkalova et al., 2018). Conservation and increase of this population, which eventually could become a source population for the restoration of leopards in the Korean peninsula, is of utmost importance.

## Supplemental Information

10.7717/peerj.8900/supp-1 Supplemental Information 1 Maximum Likelihood fits of 24 different nucleotide substitution models. Models with the lowest BIC scores (Bayesian Information Criterion) are considered to describe the substitution pattern the best. For each model, AICc value (Akaike Information Criterion, corrected), Maximum Likelihood value (lnL), and the number of parameters (including branch lengths) are also presented [1]. Non-uniformity of evolutionary rates among sites may be modeled by using a discrete Gamma distribution (+G) with 5 rate categories and by assuming that a certain fraction of sites are evolutionarily invariable (+I). Whenever applicable, estimates of gamma shape parameter and/or the estimated fraction of invariant sites are shown. Assumed or estimated values of transition/transversion bias (R) are shown for each model, as well. They are followed by nucleotide frequencies (f) and rates of base substitutions (r) for each nucleotide pair. Relative values of instantaneous r should be considered when evaluating them. For simplicity, sum of r values is made equal to 1 for each model. For estimating ML values, a tree topology was automatically computed. The analysis involved 40 nucleotide sequences. Codon positions included were 1st+2nd+3rd+Noncoding. All positions containing gaps and missing data were eliminated. There were a total of 717 positions in the final dataset. Evolutionary analyses were conducted in MEGA6 [2]. Abbreviations: GTR: General Time Reversible; HKY: Hasegawa-Kishino-Yano; TN93: Tamura-Nei; T92: Tamura 3-parameter; K2: Kimura 2-parameter; JC: Jukes-Cantor. 1. Nei M. and Kumar S. (2000). Molecular Evolution and Phylogenetics. Oxford University Press, New York. 2. Tamura K., Stecher G., Peterson D., Filipski A., and Kumar S. (2013). MEGA6: Molecular Evolutionary Genetics Analysis version 6.0. Molecular Biology and Evolution 30: 2725–2729.

10.7717/peerj.8900/supp-2 Supplemental Information 2 Bayesian tree constructed using by Beast with HKY+G+I model. KOR = Korean leopard, ORI = *P. p. orientalis*, JAP = *P. p. japonensis*, DEL = *P. p. delacouri*, KOT = *P. p. kotiya*, FUS = *P. p. fusca*, SAX = *P. p. saxicolor*, NIM = *P. p. nimr*, SHO = *P. p. pardus*, MEL = *P. p. melas*

10.7717/peerj.8900/supp-3 Supplemental Information 3 The sequences of all leopard subspecies used in this study.

## Additional Information and Declarations

### Data Availability

The following information was supplied regarding data availability: The Korean leopard NADH5/CR partial sequences are available at GenBank: MK114159 to MK114160. The Korean leopard specimen was donated to and is being stored in the Conservation Genome Resource Bank for Korean Wildlife (CGRB) at Seoul National University, Republic of Korea. This specimen can be accessed by contacting CGRB (www.cgrb.org) and submitting the application form. This sample’s registration: CGRB 15834.
