# Supplementary figures and images for: Phylogenetic study of extirpated Korean leopard using mitochondrial DNA from an old skin specimen in South Korea
Source: PeerJ. 2020 May 12;8:e8900. doi: 10.7717/peerj.8900 (PMC7227655; doi:10.7717/peerj.8900)

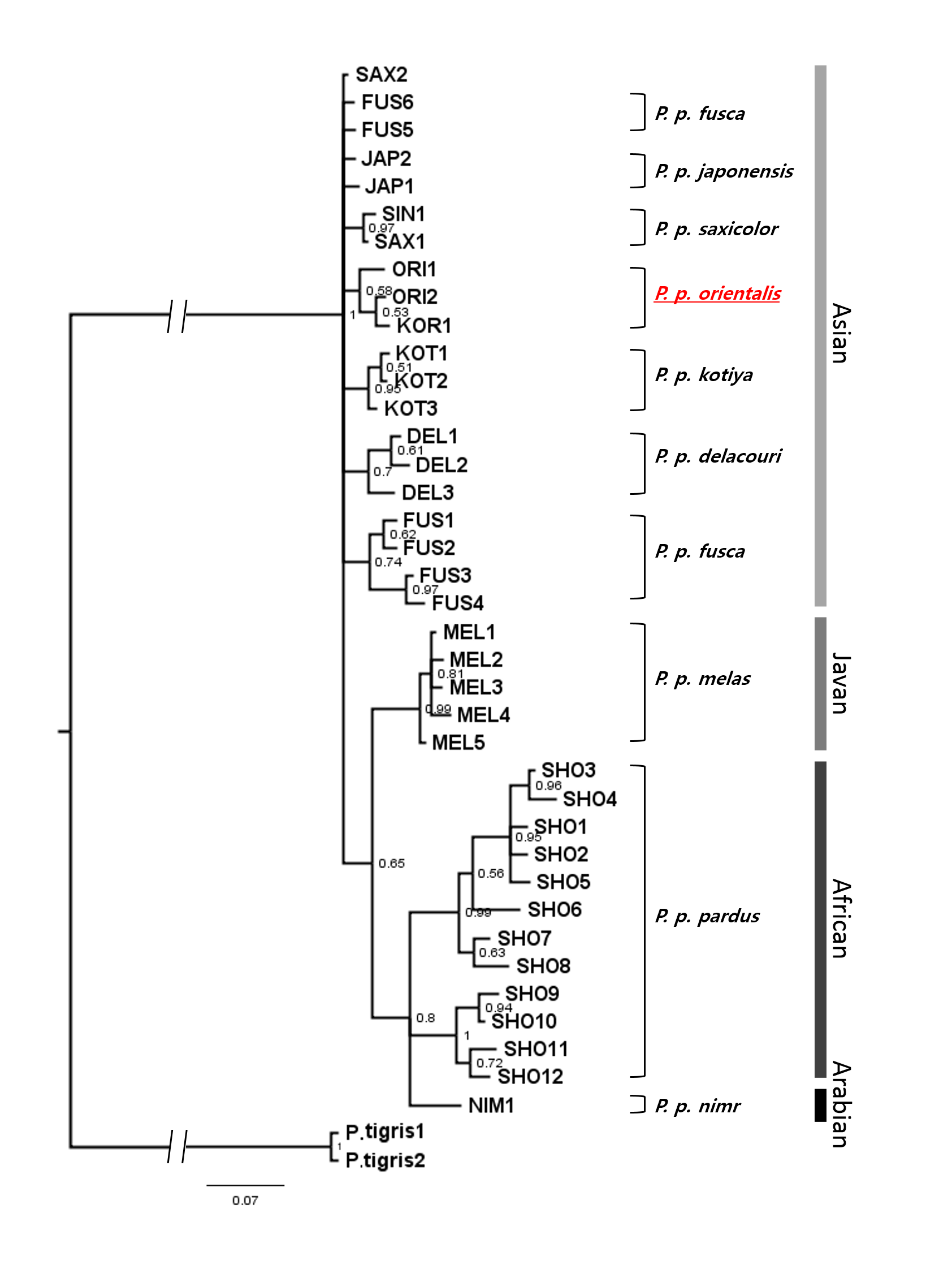

Supplement: Supplemental Information 2 — KOR = Korean leopard, ORI = P. p. orientalis, JAP = P. p. japonensis, DEL = P. p. delacouri, KOT = P. p. kotiya, FUS = P. p. fusca, SAX = P. p. saxicolor, NIM = P. p. nimr, SHO = P. p. pardus, MEL = P. p. melas [file peerj-08-8900-s002.png]
